# Development of Bio-Composites with Enhanced Antioxidant Activity Based on Poly(lactic acid) with Thymol, Carvacrol, Limonene, or Cinnamaldehyde for Active Food Packaging

**DOI:** 10.3390/polym13213652

**Published:** 2021-10-23

**Authors:** Mohammad Nahid Siddiqui, Halim Hamid Redhwi, Ioannis Tsagkalias, Evangelia C. Vouvoudi, Dimitris S. Achilias

**Affiliations:** 1 Chemistry Department, King Fahd University of Petroleum and Minerals, Dhahran 31261, Saudi Arabia; mnahid@kfupm.edu.sa; 2 Chemical Engineering Department, King Fahd University of Petroleum and Minerals, Dhahran 31261, Saudi Arabia; hhamid@kfupm.edu.sa; 3 Lab of Polymer and Color Chemistry and Technology, Department of Chemistry, Aristotle University of Thessaloniki, 54124 Thessaloniki, Greece; itsagkal@chem.auth.gr (I.T.); evouvoud@chem.auth.gr (E.C.V.)

**Keywords:** poly (lactic acid) PLA, thymol, carvacrol, limonene, cinnamaldehyde, antioxidant properties, biobased polymers, food packaging

## Abstract

The new trend in food packaging films is to use biodegradable or bio-based polymers, such as poly(lactic acid), PLA with additives such as thymol, carvacrol, limonene or cinnamaldehyde coming from natural resources (i.e., thyme, oregano, citrus fruits and cinnamon) in order to extent foodstuff shelf-life and improve consumers’ safety. Single, triple and quadruple blends of these active compounds in PLA were prepared and studied using the solvent-casting technique. The successful incorporation of the active ingredients into the polymer matrix was verified by FTIR spectroscopy. XRD and DSC data revealed that the crystallinity of PLA was not significantly affected. However, the Tg of the polymer decreased, verifying the plasticization effect of all additives. Multicomponent mixtures resulted in more intense plasticization. Cinnamaldehyde was found to play a catalytic role in the thermal degradation of PLA shifting curves to slightly lower temperatures. Release of thymol or carvacrol from the composites takes place at low rates at temperatures below 100 °C. A combined diffusion-model was found to simulate the experimental release profiles very well. Higher antioxidant activity was noticed when carvacrol was added, followed by thymol and then cinnamaldehyde and limonene. From the triple-component composites, higher antioxidant activity measured in the materials with thymol, carvacrol and cinnamaldehyde.

## 1. Introduction

The main function of food packaging is to maintain the quality and safety of food products during storage and transport and to extend their shelf life by preventing impacts from unfavorable factors or conditions, such as spoilage microorganisms, chemical contaminants, oxygen, moisture, light, and external force. Studies within the area of food active packaging are experiencing great development due to the consumers’ demand and market trends. Food active packaging systems are based on materials in which some additives with antimicrobial and/or antioxidant properties are added into the polymer matrix with the aim of extending foodstuff shelf life and improving consumer’s safety [1,2,3,4].

The demand for the use of natural additives has produced in recent years a clear increase in the number of studies based on natural extracts such as essential oils, which are categorized as ‘Generally Recognized as Safe’ (GRAS) by the US Food and Drug Administration [5], and they could be considered potential alternatives to synthetic additives, such as butylated hydroxytoluene (BHT) [6]. Essential oils extracted from plants or spices are rich sources of biological active compounds, such as terpenoids and phenolic acids [7]. The use of antioxidants additives in polymers for food packaging is a common practice to reduce their potential thermo-oxidative degradation during processing and they could prevent the food oxidation reducing the direct addition of antioxidants to the food. Among them, the great interest towards the use of renewable natural resources in the food packaging industry makes natural antioxidants candidates for stabilization of biodegradable polymer formulations.

In particular, carvacrol and thymol are present as major compounds in thyme and oregano essential oils [8]. These two compounds are phenolic monoterpenes and isomers that exhibit significant in vitro antibacterial activity [9]. Carvacrol shows antifungal, insecticidal, antitoxigenic and antiparasitic activities [10]. On the other hand, thymol has received considerable attention as an antimicrobial agent, showing very high antifungal activity, also being an excellent food antioxidant [11,12]. The antimicrobial activity of both compounds has been already studied and reported against several bacterial strains [13]. D-limonene is a monoterpene obtained from a variety of citrus fruit peel oil which is the most important residue in citrus industry. It is food grade and presents antioxidant properties. Another compound reported to exhibit antimicrobial properties is cinnamaldehyde coming from cinnamon.

The term ‘active’ or ‘intelligent’ packaging was first introduced by Regulation 2004/1935/EC of the European Parliament and Council [14]. Natural extracts such as essential oils and their constituents such as carvacrol, thymol or limonene are categorized as flavorings by European Commission Decision 2002/113/EC. In this framework, the formulation of active packaging systems with controlled migration of natural antioxidants and/or antimicrobial compounds to foodstuff might result in prolonged shelf life and preservation of quality of the product. Therefore, the incorporation of compounds such as thymol, carvacrol, limonene or cinnamaldehyde into polymeric materials intended to be in contact with foodstuff is an attractive option for consumers, as well as for packaging manufacturers.

One of the major problems with plastic materials is that they are not biodegradable, meaning that most have a life span of over 100 years. One way to overcome this problem while retaining the benefits of plastics is to use biodegradable materials. Biodegradable polymers, mainly polyesters, can be easily recycled by composting and degradation over a period from a few months to 1 year [15,16]. Poly(lactic acid), PLA, is such a biodegradable, biocompatible and compostable polyester derived from renewable resources such as corn, potato, cane molasses and beet sugar [17,18,19,20,21]. High molecular weight PLA has characteristics and properties similar to those of commodity polymers, such as polypropylene (PP), polyethylene (PE), or polystyrene (PS), and therefore might replace these polymers’ innumerous applications [22]. PLA can be produced with existing manufacturing equipment (that is designed and originally used for petrochemical-derived plastics), making it relatively cost effective to produce. Accordingly, PLA has the second largest production volume of any bioplastic (the most commonly cited is thermoplastic starch) [23]; is the most promising environmentally friendly thermoplastic [24]; and has promising applications in packaging, consumer goods, fibers and biomedicine because of its excellent mechanical properties, transparency and biosafety [25,26,27,28]. Additionally, the ease with which PLA melts allows for some interesting applications in 3D printing is also significant. Therefore, PLA has attracted great research interest in the scientific community. Therefore, PLA was selected to be studied here based on specific criteria, including its commercially availability, research interest, compostability and safety to be used in food packaging applications.

The development of biocomposite films based on PLA incorporating thymol as the active additive was proposed by Ramos et al. [29,30]. Thermal stability was not significantly affected by the addition of thymol, whereas the PLA glass transition temperature was decreased, as the result of the polymer plasticization, and the elastic modulus and elongation at break were modified. The antioxidant activity produced by the 2,2-diphenyl-1-picrylhydrazyl (DPPH) spectroscopic method suggested that the formulated nano-biocomposites could be considered a promising antioxidant active packaging material. Stepczyńska, 2019, presented biodegradable materials, which exhibit biocidal activity against microorganisms due to the application of natural vegetable compounds, [31]. The effect of PLA modification, carried out by using bioactive compounds such as: carvacrol and eugenol, thymol and eugenol, thymol and carvacrol as mixtures, on the thermal properties and biodegradation rate of this material was determined. The use of active compounds in mixtures gives a synergistic effect. Several other authors prepared films of PLA with thymol or carvacrol and studied the physicochemical, thermal and morphological properties of the polymer [32]. PLA crystallinity and rigidity were found to slightly decrease when these compounds were added [33,34,35,36]. Composites were prepared by either the melt-blending or the solvent-casting method. It was observed that the addition of both thymol and carvacrol reduced the glass-transition temperature of PLA, and slightly decreased the maximum degradation temperature. The antioxidant capacity of PLA fibers varied from 53 to 65% for 5–20% carvacrol content [37,38]. Membranes based on PLA with carvacrol were prepared by Scaffaro et al. [39,40,41] via electrospinning. The results revealed that carvacrol has good compatibility with PLA and acts as a plasticizer, improving flexibility and extensibility of the matrix. 

Moreover, the antioxidant activity of either thymol or carvacrol in PLA-based films was studied as a means to extend the shelf life of several foods, including salmon slices [42], lettuce [43,44], ground beef [45] and blackberries and raspberries [46].

Limonene was used mainly as a plasticizer in composites based on PLA for food packaging applications [47,48,49,50]. The addition of limonene into the PLA matrix was found to reduce, as expected, the glass transition temperature of the films, and to affect the mechanical properties of the films, increasing the plastic response of PLA. 

Sangkasanya et al., 2018 [51] prepared a new plastic packaging material to prolong the shelf life of fresh beef meat during cold storage by blending PLA with various concentrations (0, 4, 6 and 8%, *w*/*w*) of limonene. These results indicate that the PLA containing limonene can be utilized as an antimicrobial packaging for the beef meat.

Furthermore, the antimicrobial properties of cinnamaldehyde when added to PLA were studied by several authors [52,53,54] The physicochemical and microbial quality of button mushrooms (*Agaricus bisporus*) stored at 4 ± 1 °C for 16 days in this packaging was investigated. The best compromise between mechanical, barrier, thermal, and antimicrobial properties could be achieved by the addition of 9 wt% cinnamaldehyde into PLA/PTMC blends. To maintain the quality of postharvest fruits continuously and meet the health requirements of consumers, a high barrier and long-lasting antibacterial PLA film with included cinnamaldehyde as packaging material was developed by Zhang et al. [55]. Muller et al., 2017 [56,57] used the solution casting technique to prepare PLA films with cinnamaldehyde, using ethyl acetate as solvent. The release kinetics of the active compound in different food simulants demonstrated that a part of cinnamaldehyde was tightly bonded to the PLA matrix, whereas the free compound diffused more easily. Cinnamaldehyde was found to have a plasticizing effect on the PLA matrix. [54,58,59].

Recently, the synthesis of d-limonene-loaded polymeric nanoparticles with enhanced antimicrobial properties [60] and the effect of montmorillonite/essential oil nanohybrids on the kinetics of the in situ radical polymerization of styrene [61] were also investigated by our laboratory.

From the above extended literature survey, the main conclusions are: there has been significant recent research on the addition of thymol and carvacrol (mainly) but also on the addition of limonene and cinnamaldehyde to PLA in order to produce biocomposite films with advanced and active properties. The materials formed usually exhibit enhanced antioxidant and antimicrobial properties, whereas most of these compounds act as plasticizers. However, studies on the combinatorial action of these compounds on PLA properties or a direct comparison of their activity are very scarce, if any. As a step further, the aim of this research was to investigate the physicochemical, thermal and antioxidant properties of PLA films loaded with the naturally occurring compounds thymol, carvacrol, limonene or cinnamaldehyde and their triple or quadruple mixtures. Therefore, it would be interesting to study if synergistic effects take place. According to our knowledge, this is the first time that the effect of loading triple or quadruple mixtures of these compounds to PLA has been examined regarding their final material thermal and antioxidant properties. 

## 2. Materials and Methods

### 2.1. Materials

The additives used were thymol (purity > 99%, CAS: 89-83-8, Sigma-Aldrich, Darmstadt, Germany), carvacrol (purity > 99%, CAS: 499-75-2, Sigma-Aldrich, Darmstadt, Germany), cinnamaldehyde, (purity > 95%, CAS: 104-55-2, Sigma-Aldrich, Darmstadt, Germany) and D-limonene (purity ≥ 99%; M = 136 g/mol, b.p. = 176–177 °C, d = 0.841; Fluka, Fisher Scientific, Leicestershire, UK). Film grade PLA Ingeo (Mw = 185,000 g/mol) was obtained from Nature Works LLC, Minnetonka, MN, USA and used as received. The solvents used were THF (HPLC grade), DMF, methanol and dichloromethane, all from Sigma-Aldrich, Darmstadt, Germany. All solvents were used with no further purification.

### 2.2. Experimental Procedure

PLA-based nanocomposites containing different relative amounts of thymol, carvacrol, cinnamaldehyde and limonene were synthesized. In order to investigate the effect of different amounts of the four compounds as well as their possible synergistic effect on the properties of the composites formed, the following design of experiments was carried out (Table 1). Initially, each compound was added separately in two loadings (i.e., 5 and 10 wt%) and subsequently four triple mixtures were prepared with all different combinations and a quadruple with all four components. In all mixtures, an equal amount of each component was added.

The PLA-based films were synthesized using two different techniques, i.e., melt-mixing and solvent casting. In the melt-mixing technique, a screw extruder was used. However, this method presented various problems, mainly due to the evaporation of volatile materials caused by the high temperatures employed to melt the polymer, PLA. Thus, the solvent casting technique was finally procured and used in the formulation of all materials studied here. Several solvents were tested to dissolve the polymer and the additives used. Tetrahydrofuran (THF) was found to be an adequate solvent for this purpose. Accordingly, the polymer PLA was dissolved in THF (2 g per 30 mL of solvent) in a cylindrical flask and using a magnetic stirrer for almost 30 min. Subsequently the required amount of the additive was added, and the mixture was agitated for another 30 min. After complete mixing (no visible phase separation) the mixture was transferred to a slab to evaporate the solvent. Films were considered to be ready when no variation of their mass with time was recorded.

### 2.3. Quantification of the Active Ingredients in the PLA Matrix

The actual amount of active ingredients (i.e., thymol, carvacrol, limonene and cinnamaldehyde) in PLA films after processing was determined by solid–liquid extraction followed by high-performance liquid chromatography coupled to ultraviolet spectroscopy (HPLC-UV) analysis, according to the procedure proposed by Ramos et al. [29]. Initially, 50 ± 8 mg of each film were extracted with 10 mL of methanol in an amber vial at 40 °C for 24 h. The amount of active ingredients was determined using a Shimadzu Prominence HPLC system (Kyoto, Japan) consising of a degasser (Model DGU-20A5), a pump (Model LC-20AD), a manual injector with a 100 μL loop (Model Rheodyne, Cotati, CA, USA), a column oven (Model CTO-20AC) and a UV detector at 275 nm. The column used was a LiChrospher 100 RP 18 (250 mm × 5 mm × 5 μm, Agilent Technologies, Santa Clara, CA, USA). The mobile phase was composed of acetonitrile and water (40:60) at a 1 mL/min flow rate. The entire HPLC analysis was performed using LC solutions software, v. 1.21 SP1 (Shimadzu Corporation, Kyoto, Japan). An amount of 20 μL of the extracted samples were injected and analyses were performed in triplicate. Quantification of the active additives was carried out by comparison of the chromatographic peak areas with standards in the same concentration range. Calibration curves were run at five concentrations from 100 to 500 mg/kg using appropriately diluted standards of either thymol, or carvacrol, or limonene or cinnamaldehyde in methanol. The amount of the compound released at 40 °C after 24 h was considered the entire active ingredient contained in the films. A similar procedure has been used by other authors in the literature [62,63]. The amount of the active compounds released into the methanol was compared with the amount incorporated into the film-forming solution to calculate the amount loss during the drying step. Specifically, the loading capacity (*LC*) was defined by the ratio of the amount of the active ingredient detected in the formulation, *W_t_*, over the total amount of the polymer, *W_p_* and the encapsulation efficiency (*EE*%) was defined by the ratio of the amount of the active ingredient detected in the formulation, *W_t_*, over the initial amount of the active agent used to make the formulation, *W_i_*, using the below formula:

(1) $$LC=\frac{W_{t}}{W_{p}}\times 100\;\;;\;\;EE=\frac{W_{t}}{W_{i}}\times 100$$

### 2.4. Analytical Techniques

*Fourier-Transform Infra-Red (FTIR).* The chemical structure of the neat PLA and the incorporation of the additives, i.e., thymol, carvacrol, limonene and cinnamaldehyde in PLA was confirmed by recording their IR spectra. The instrument used was the Spectrum 1 spectrophotometer from Perkin Elmer with an attenuated total reflectance (ATR) device. Measurements were carried out using thin films prepared in a hot hydraulic press and spectra recorded over the range from 4000 to 600 cm^−1^ at a resolution of 2 cm^−1^, and 32 scans were averaged to reduce noise. The instrument’s software was used to identify several peaks.

*Differential Scanning Calorimetry (DSC)*. In order to estimate the glass transition temperature of every material prepared, the DSC-Diamond (Perkin-Elmer) was used. Approximately 5–6 mg of each sample were weighed, put into the standard Perkin-Elmer sample pan, sealed and placed into the appropriate position of the instrument. Subsequently, they were initially heated to 200 °C at a rate of 10 °C min^−1^. Following this, the samples were cooled to 20 °C and their glass transition temperature was measured by heating again to 200 °C at a rate of 10 °C min^−1^. All results are from the second heating. 

*X-ray diffraction (XRD).* X-ray powder diffraction (XRD) patterns of the neat PLA and all composite materials were recorded using an XRD-diffractometer (Rigaku, model MiniFlex 600, Chalgrove, Oxford, UK) with CuKa radiation for crystalline phase identification. The samples were scanned from 5° to 30°.

*Pyrolysis/Gas-Chromatography*. Py/GC-MS was performed in a GC-MS composed of a QP-2010 Ultra Plus chromatographer (Shimadzu, Kyoto, Japan) and a QP-2010 Ultra mass spectrometer (Shimadzu, Kyoto, Japan), equipped with an EGA/PY-3030D multi-shot pyrolizer (Frontier Laboratories, Kyoto, Japan). The program run during the EGA measurements included heating from 50 to 550 °C at 20 °C/min under the flow of He as a purge gas.

*Thermogravimetric Analysis (TG).* In order to measure the release rate profiles of the additives from the composites, isothermal experiments were carried out at several temperatures using thermogravimetric analysis. TG was performed on a Pyris 1 TGA (Perkin-Elmer) thermal analyzer. For the experiments, approximately 20 mg samples were weighed and placed inside the TGA pan sample holder. The samples were rapidly heated to the desired temperature and remained for a prespecified time at this temperature under nitrogen flow. The amount of the active compound released was estimated after subtracting from the initial mass of the sample the mass continuously recorded.

*Antioxidant activity.* The antioxidant activity was evaluated, using the DPPH (2,2-diphenyl-1-picrylhydrazyl) test. Accordingly, 6 mg of each film was put into a glass vial containing 3 mL DPPH solution and incubated at 25 °C for 1, 3 and 20 h in darkness. The reaction kinetics were followed by the disappearance of the DPPH• reactant as given by the absorbance measurement from 400 to 700 nm (max. absorbance: 516 nm) using UV spectrophotometer (Shimadzu Spectrophotometer UV-1800). The percent antioxidant activity (AA) of the films was calculated by measuring the absorbance (ABS) at 516 nm taking the absorbance of the DPPH solution as control, according to the following equation:

(2) $$AA(\%)=\frac{ABS_{control}-ABS_{sample}}{ABS_{control}}\times 100$$

### 2.5. Mathematical Modeling of the Release Rate Profiles

In order to study the kinetics of the release of the active compounds from the PLA-based films, different diffusion-based mathematical models were employed. Results of the models were fitted to the normalized volatile active compound desorption data (as derived from the isothermal TGA analysis) to estimate the desorption rate and diffusion coefficients. A Generalized Reduced Gradient Nonlinear Solving Method for nonlinear optimization was used to fit the nonlinear model to the experimental data, while a Chi-squared test was used to assess the goodness of fit.

## 3. Results

### 3.1. Quantification of the Active Ingredinets in the PLA Matrix and Characterization of the Chemical Structure of the Composites Prepared

The amount of thymol, carvacrol, limonene or cinnamaldehyde present in the formulations after processing appear in Table 2 in terms of loading capacity and encapsulation efficiency as defined in Equation (1). Results showed that using the melt-mixing technique, the encapsulation efficiency is near 70%, meaning that almost 30% of the active ingredient was lost during processing by evaporation due to the high temperature used during polymer melting [29]. In contrast, using the solution casting technique, the estimated amount of the encapsulation efficiency of thymol, carvacrol and cinnamaldehyde were always high and found to vary from 94 to 98%. Slightly lower values were estimated for limonene, i.e., 90 to 92%. It was thus verified that the amount of active compounds evaporated during this process was small. This is due to the low temperature used during the evaporation (i.e., 25 °C), since the boiling point of the solvent used (i.e., THF) was 66 °C, much lower compared to those of thymol, carvacrol, cinnamaldehyde and limonene, (i.e., 233, 237, 248 and 178 °C, respectively) and the low vapor pressure of these compounds at this temperature (i.e., 8.5 × 10^−5^, 5.4 × 10^−5^, 2.6 × 10^−3^ and 4.1 × 10^−5^ bar for thymol, carvacrol, limonene and cinnamaldehyde) compared to that of THF, i.e., 0.215 bar. Encapsulation efficiencies higher than 94% for all active ingredients were also measured in the triple and the quadruple blends.

**Figure 1:**
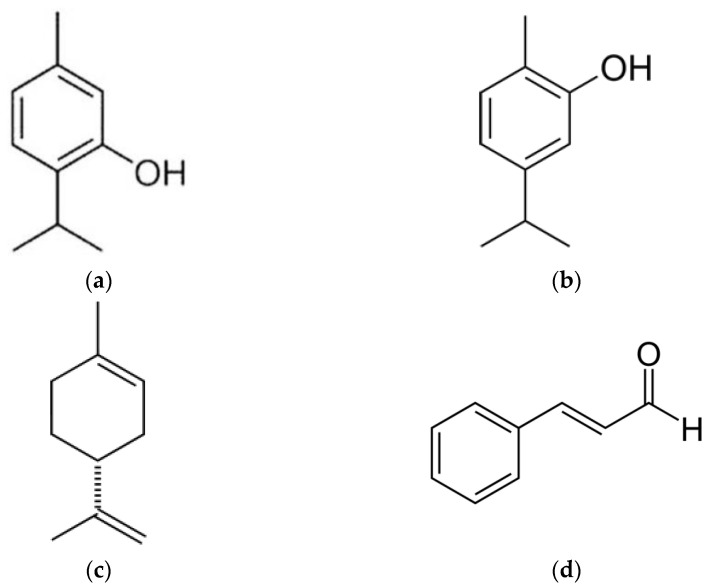
Chemical structure of (**a**) thymol, (**b**) carvacrol, (**c**) limonene and (**d**) cinnamaldehyde.

**Figure 2:**
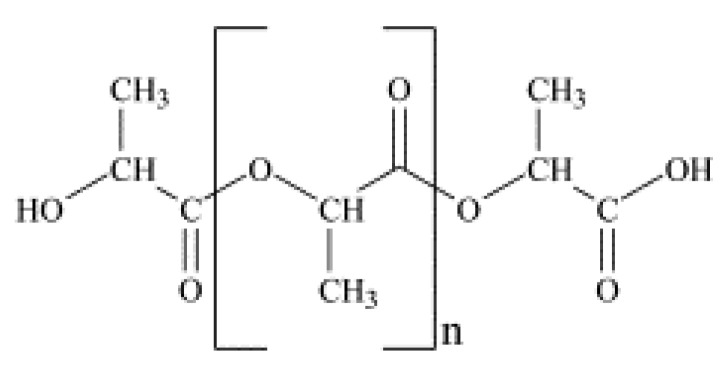
Chemical structure of the repeating unit of PLA.

The chemical structure of the additives used and the repeating unit of the polyester, PLA, are shown in Figure 1 and Figure 2, respectively. 

**Figure 3:**
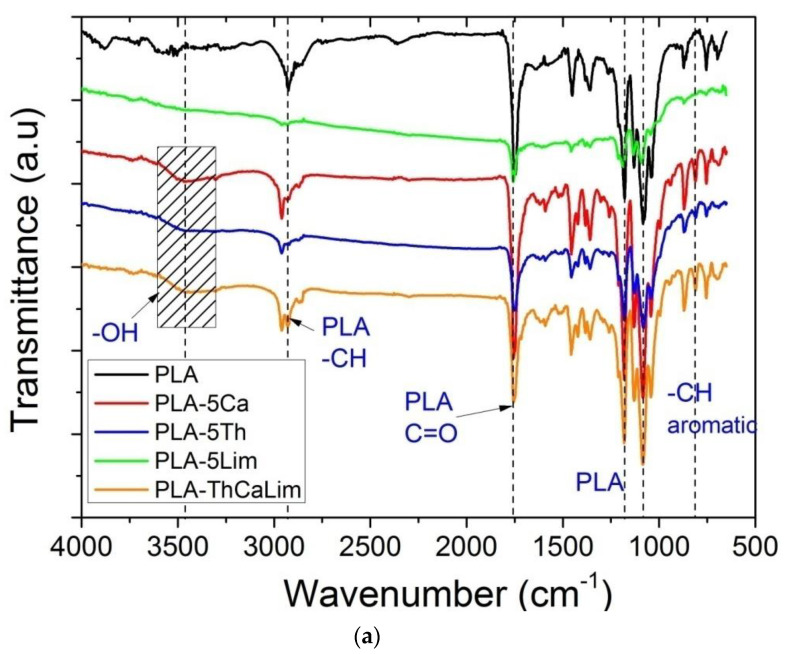

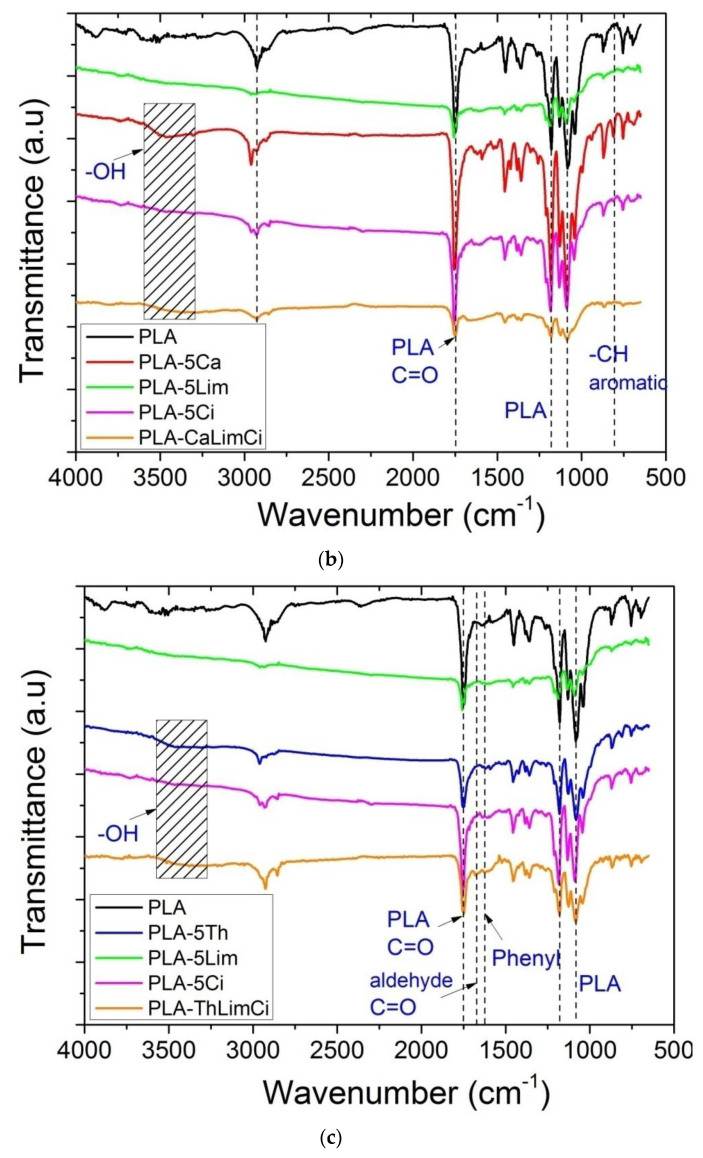

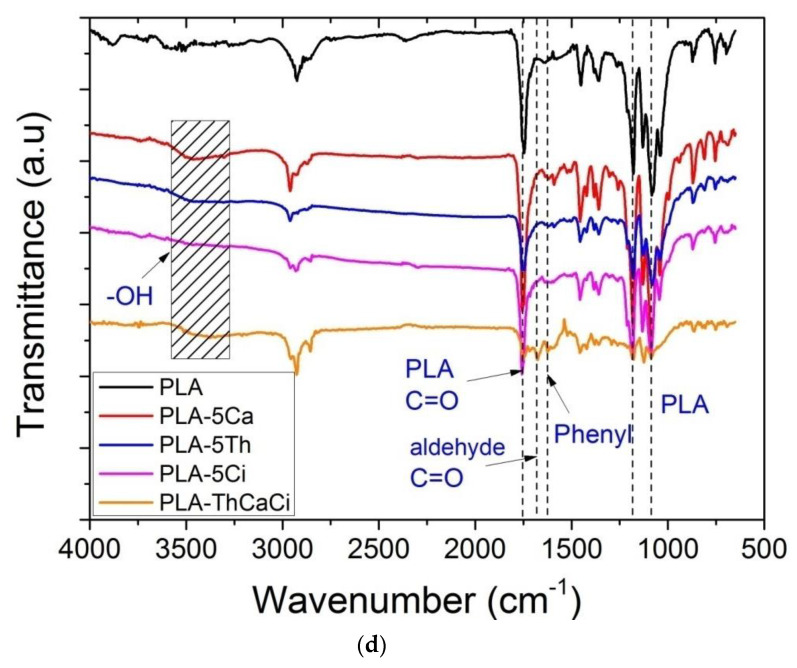
FTIR spectra of neat PLA and the triple blend of PLA with thymol, carvacrol and limonene, PLA-ThCaLim, together with the corresponding spectra of PLA with each of the active ingredients (**a**) of neat PLA and the triple blend of PLA with carvacrol, limonene and cinnamaldehyde, PLA-CaLimCi, together with the corresponding spectra of PLA with each of the active ingredients (**b**) of neat PLA and the triple blend of PLA with thymol, limonene and cinnamaldehyde, PLA-ThLimCi, together with the corresponding spectra of PLA with each of the active ingredients (**c**) and of neat PLA and the triple blend of PLA with thymol, carvacrol and cinnamaldehyde, PLA-ThCaCi, together with the corresponding spectra of PLA with each of the active ingredients (**d**).

**Figure 4:**
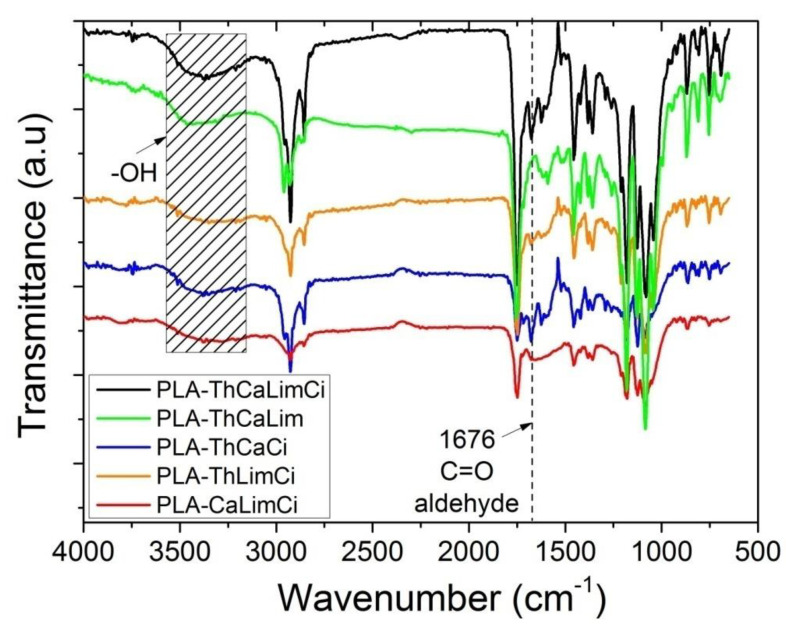
FTIR spectra of the PLA composites with all triple mixtures and the quadruple mixture of thymol, carvacrol, limonene or cinnamaldehyde.

Fourier transform infrared (FTIR) spectroscopy was used for the identification of the characteristic chemical groups of the composites prepared. FTIR spectra of PLA with all combinations of the triple blends together with the spectra of the corresponding material having the individual components and neat PLA are illustrated in Figure 3a–d. Furthermore, in Figure 4 appear the FTIR spectra of all triple and the quadruple composites of PLA with carvacrol, thymol, cinnamaldehyde or limonene.

The most characteristic peaks of the neat PLA are at: 2927 cm^−1^, attributed to symmetrical and asymmetrical –CH bond stretching of the group –CH_3_; 1751 cm^−1^, attributed to the carbonyl C=O stretching, the bands in the range 1300–1500 cm^−1^ (mainly 1358 and 1452 cm^−1^) are assigned to symmetric and asymmetric deformational bending vibrations of C–H present in CH_3_ of PLA, and the peaks at 1082 and 1180 cm^−1^ are attributed to C–O–C stretching of PLA. 

The incorporation of thymol or carvacrol into the PLA films was confirmed by the presence of a clear broad strong band ranging from 3300 to 3600 cm^−1^ and peaking near 3450 cm^−1^ attributed to the stretching vibrations of the phenolic hydroxyl groups –OH present in both these compounds. This absorption band was a clear proof and a good indication of the success of the preparation of the composites. Aromatic C–H bending is clear at 812 cm^−1^. A peak at 1596 cm^−1^ appeared also in thymol and carvacrol due to the existence of an aromatic ring also in these compounds attributed to vibrations of C=C.

The incorporation of cinnamaldehyde into the PLA films was confirmed by the presence of the carbonyl (C=O) stretch peak of the aldehyde in cinnamaldehyde at 1668 cm^−1^. This peak in our composites showed a slight shift to higher frequency, i.e., 1672 cm^−1^. This observation suggests that cinnamaldehyde formed some hydrogen bonds between the aldehyde –H and the ester carbonyl C=O group. Furthermore, a peak at 1624 cm^−1^ is ascribed to aromatic C=C vibrations of the phenyl ring and the alkene groups are shown at 1458 cm^−1^.

The incorporation of limonene into the PLA films was confirmed by the presence of a peak at 1452 cm^−1^ attributed to –CH_2_ deformation mode, peaks at 1032 and 1082 cm^−1^ attributed to CH out of the plane vibrations and the out of the plane vibration at 863 cm^−1^ due to =CH_2_. A peak around 1650 cm^−1^ is related to the stretching of the double bonds (C=C) found in the endocyclic and exocyclic positions of the structure of limonene.

**Figure 5:**
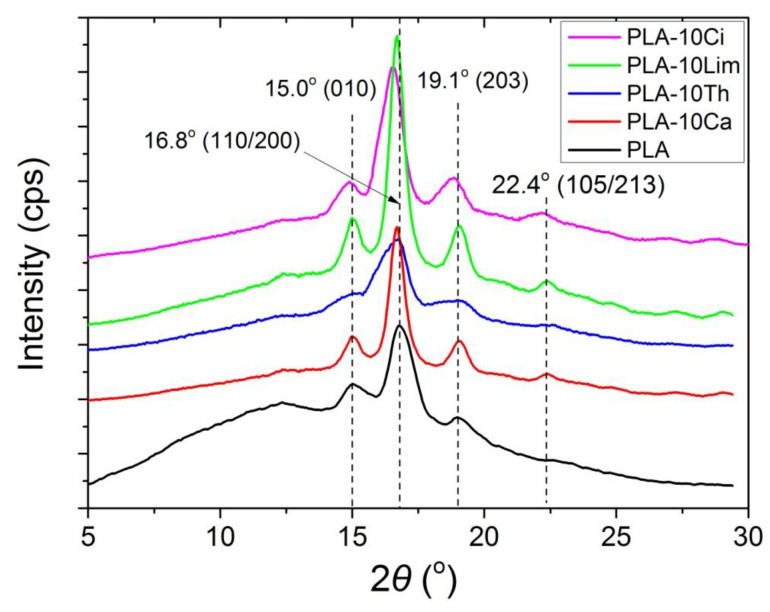
X-ray diffraction (XRD) patterns of the pure PLA and their composites with 10% thymol, carvacrol, limonene or cinnamaldehyde.

The crystal structure and morphology of the semi-crystalline polyester PLA have been extensively investigated in the literature using many techniques [64,65]. X-ray diffraction is one of the most accurate and representative techniques. It has been reported that PLA crystallizes in several forms, the formation of which depends on the crystallization conditions. The most common α-form occurred in conventional melt and solution crystallization conditions. Therefore, to evaluate the crystal morphologies of pure PLA and their composites, wide-angle X-ray diffraction measurements were performed. Figure 5 shows the XRD patterns of neat PLA and its composites with thymol, carvacrol, limonene and cinnamaldehyde. Neat PLA exhibits a very strong diffraction peak at 2*θ* = 16.8° due to diffraction from (110) and/or (200) planes and the less intense peaks at 2*θ* = 15.0 and 19.1°, attributed to reflections of the (010) and (203), respectively. Finally, a shoulder is clear at 22.4°, attributed to reflections of the (105/213) planes. All these are representative peaks of the α-form crystals, indicating that PLA has the typical orthorhombic crystal structure. Similar peaks have been reported in the literature for PLA [64,65].

Furthermore, from this figure it can be seen that the incorporation of thymol, carvacrol or limonene does not alter the crystal structure of the PLA matrix, whereas a slight shift to lower angles was observed when cinnamaldehyde was added. 

### 3.2. Thermal Properties

**Figure 6:**
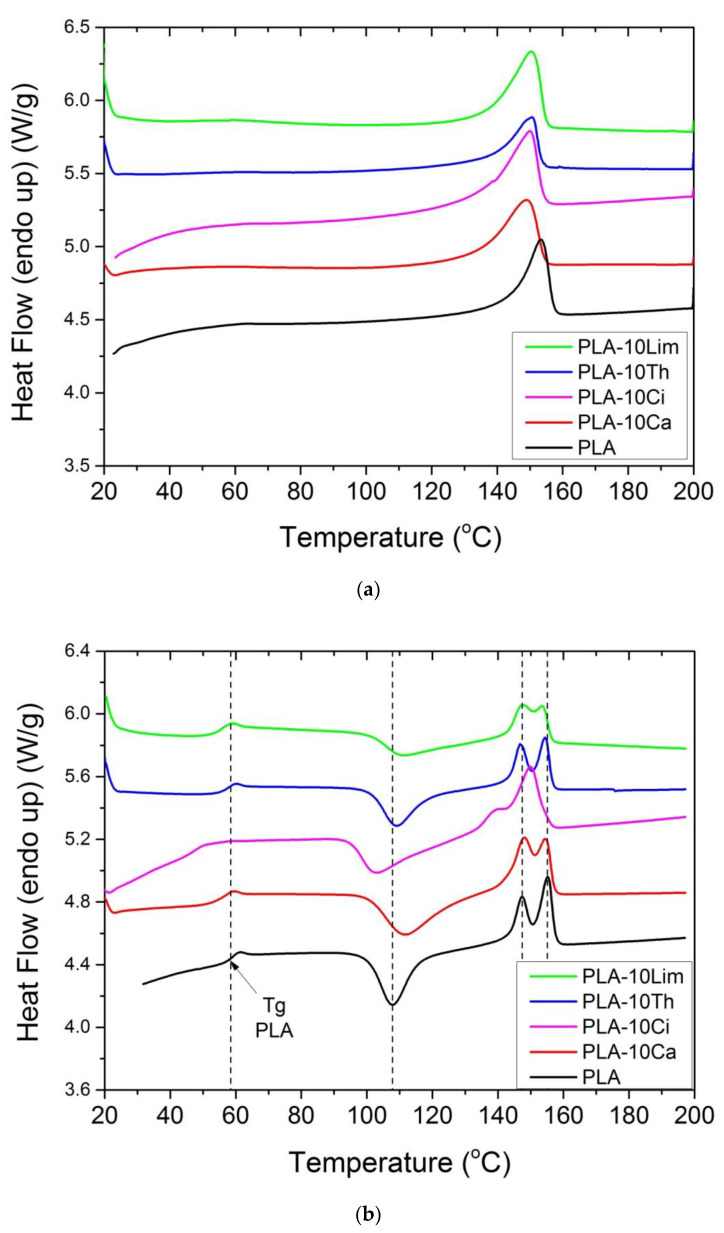
DSC measurements of PLA and their composites with 10 wt% thymol, carvacrol, limonene or cinnamaldehyde recorded during the first (**a**) or the second heating (**b**).

The thermal behavior of the films prepared by the solution casting technique was investigated by Differential Scanning Calorimetry (DSC). Figure 6a shows the results of the DSC traces after the first heating, while in Figure 6b the corresponding measures after the second heating are included. From the first-heating scans, the glass transition temperature of the polymer was not clear. All samples presented a single melting peak at 152.5 °C for neat PLA and near 150 °C for all other composite materials. No cold crystallization was observed, which is common for PLA films obtained after solution casting [64]. However, in order to omit the influence of the synthesis conditions and investigate only the influence of the additive on the thermal properties of the PLA, results from the second heating scans were only evaluated. As shown in Figure 6b, three main transitions are observed successively: a glass transition (along with an enthalpy relaxation peak), a cold crystallization exotherm and a melting endotherm. All results are included in Table 3.

**Figure 7:**
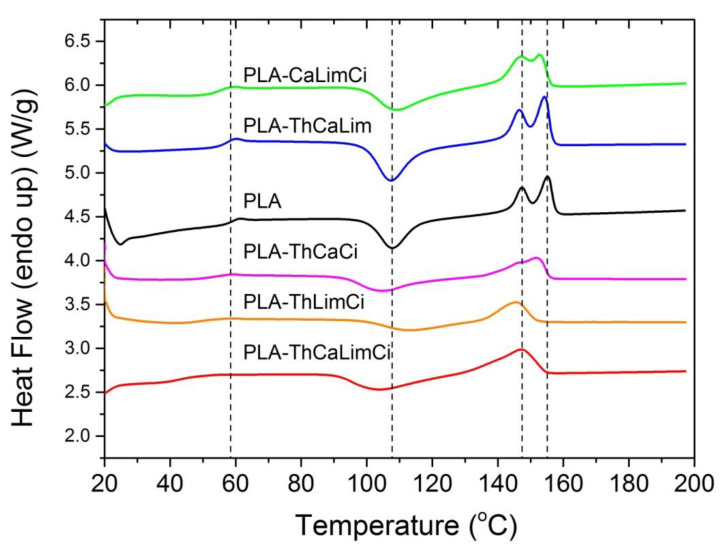
DSC scans of PLA and their composites with the triple or quadruple blends.

It could be seen that pure PLA exhibited a glass transition temperature at 58.4 °C. The addition of all compounds resulted in a reduction of Tg, which was near 55–56 °C in the composites with thymol, carvacrol or limonene and even lower, near 50 °C in the case of adding cinnamaldehyde. Thus, the plasticization effect of all compounds on the PLA matrix was verified. Additionally, the DSC scans of the composites obtained after adding different combinations of the compounds are illustrated in Figure 7 and results are included in Table 3. It was clear that, concerning the Tg of the polymer, the combination of the additives resulted in much lower values and therefore more intense plasticization. Particularly, Tg was reduced to 50–56 °C in the triple compound mixtures and nearly 9 °C, to 49.4 °C when all four components were added. 

The absolute degree of crystallinity (*Xc*) of the samples was evaluated from the heat which evolved during crystallization by the following relationship (Equation (3)) [64]

(3) $$X_{C}(\%)=\frac{\Delta H_{f}}{w_{PLA}\times \Delta H_{f}^{0}}\times 100$$

where *Xc* is the degree of crystallinity of the PLA, Δ*H_f_* is the heat of fusion of the PLA in the blend, Δ*H_f_^0^* is the heat of fusion for 100% crystalline PLA (93 J/g) [66] and *W_PLA_* is the weight fraction of PLA in the blend. 

The values thus obtained are included in Table 3. The degree of crystallinity estimated for neat PLA was near 30% and did not change significantly when all different compounds were added; this means that, according also to the XRD results, the crystallinity of the samples was not affected by the presence of the additives.

From Figure 6b, it can be seen that pure PLA and all composites exhibited clear cold crystallization. The temperature measured for neat PLA was near 108 °C, whereas those of all other materials ranged slightly above or below this value, from 103 to 111 °C. Moreover, a bimodal melting peak was noted for all samples. The two peaks appear in two temperature ranges: T_m1_ = 146–148 °C and T_m2_ = 152–155 °C and are related to two different families of crystals, which may differentiate by crystal perfection. It could be seen from the Figure 6b that the first melting peak was not significantly affected by the presence of the additive, whereas the second slightly decreased. This is an indication that the addition of the additives slightly promoted the formation of a’-form crystals. In the case of the material with all four components added, the two peaks were merged into one. The effect of adding the additives on the ability of PLA to crystallize was further checked by measuring the heat of fusion (Δ*H_f_*) (Table 3). The values measured were lower but if they are divided by the real amount of the polymer then it seems that the crystallinity of PLA was not significantly affected. 

**Figure 8:**
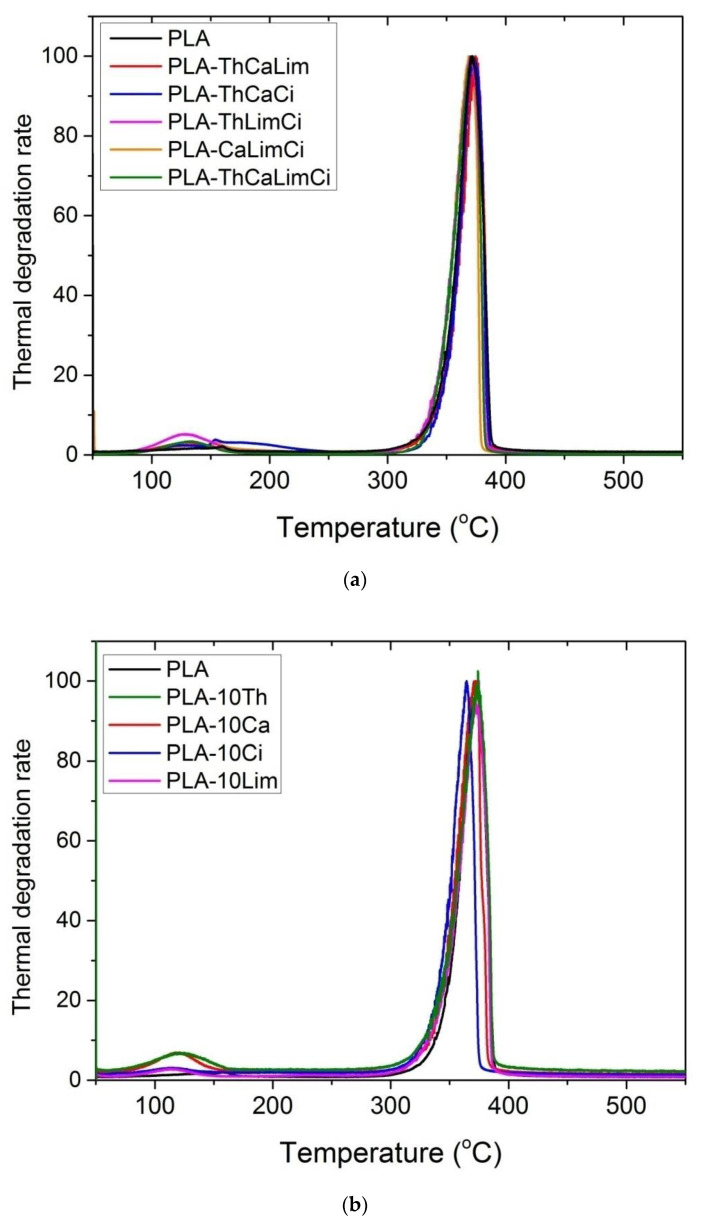
Thermal degradation under an inert atmosphere of PLA and its composites with a single additive (**a**) or ternary or quaternary composites (**b**) obtained from a pyrolizer using the EGA method.

Figure 8 presents the curves of all composite materials based on PLA obtained from the pyrolyzer running under the EGA program. Thus, information on the thermal stability of the films could be derived. It can be seen that neat PLA and all materials exhibit a single degradation curve peaking at nearly 372 °C. Thermal degradation starts near 319 °C and ends at almost 388 °C. Addition of thymol, carvacrol and limonene to PLA did not seem to cause any significant change in the temperature of the maximum degradation rate (T_max_) and the whole curve. It means that all PLA composites, except those with cinnamaldehyde, had similar thermal stability. However, addition of cinnamaldehyde seems to shift the curve to lower values with the peak temperature being almost 7 °C lower, from 372 to 365 °C. Similar behavior was observed in the three-part composites when cinnamaldehyde was included. Although, the shifting in temperature was lower (nearly 3 °C) at the maximum degradation rate, since the amount of cinnamaldehyde added was lower compared to the material including only cinnamaldehyde. Thus, it seems that cinnamaldehyde has a role as a possible catalyst of the thermal degradation of PLA. It is probable that the double bond in the chemical structure of this compound breaks at these temperatures, resulting in the production of radicals that could enhance the degradation of the polymer. However, this observation needs further investigation. A small peak appearing near 120 °C seems to be due to possible volatilization of the additive, which is examined in more detail in the next section. 

### 3.3. Release Profiles of the Active Compounds from the Polymer Matrix and Study of the Process Kinetics

**Figure 9:**
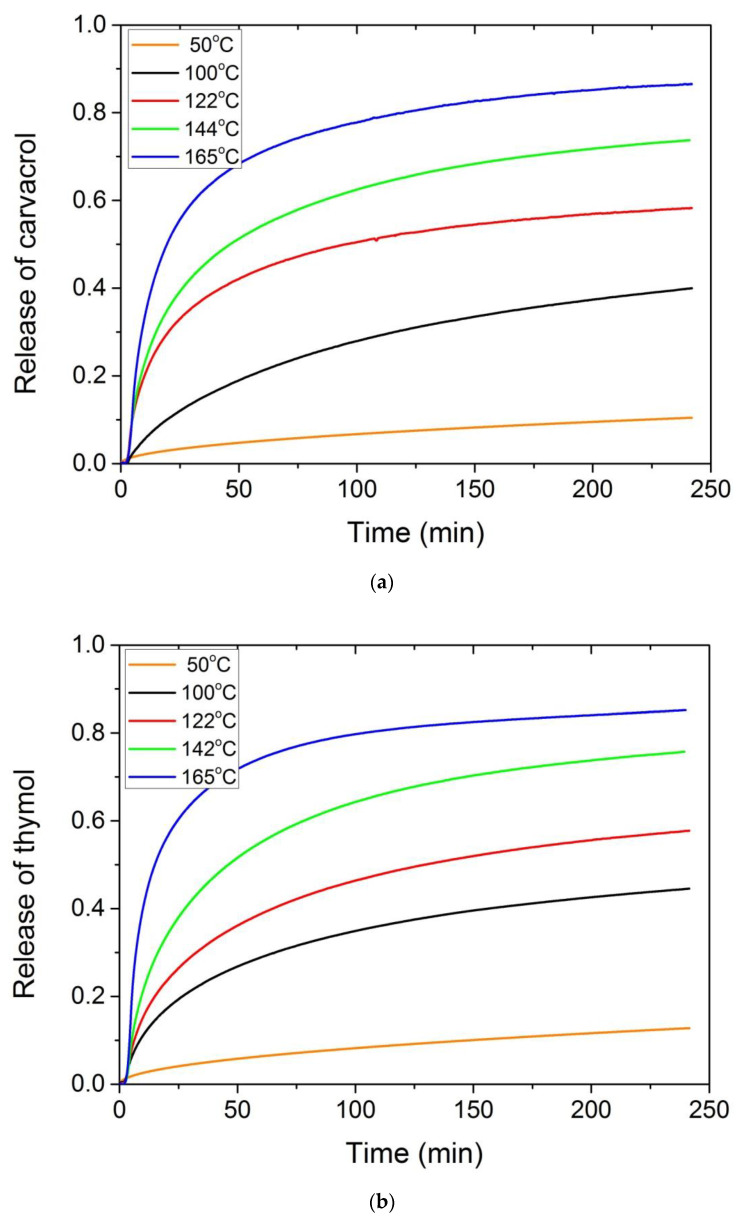
Release profiles of carvacrol (**a**) or thymol (**b**) from the polymer matrix at five different temperatures, 50, 100, 122, 144 and 165 °C. Measurements were carried out isothermally using TGA.

Furthermore, in order to check the small degradation peak observed in the cases of the composites with thymol and carvacrol, release profile studies were performed for these two materials at different temperatures. Particularly, the release of the active component thymol or carvacrol from the polymer matrix was studied at different temperatures using isothermal TGA scans. Five temperatures were selected, i.e., 50, 100, 122, 144 and 165 °C. Results appear in Figure 9a,b for carvacrol and thymol, respectively.

From the active component, release tests the following results are obtained: The amount released is definitely dependent on temperature, with higher temperatures resulting in faster release rates. At 100 °C it takes almost 4 h for the release of 40% of the initial amount, whereas at 122 °C almost the half of the initial amount is released after 4 h. At low temperatures, there is a constant but small amount of the active compound released. Therefore, treatment of these samples at high temperatures for prolonged time is not recommended since most of the active compound will be released in less than 1 day.

Loss of the active compound from the polymer matrix is a process governed by diffusion. To study the mechanism that rules the release of thymol or carvacrol from the synthesized films, the release data obtained by isothermal TGA measurements of the blends at different temperatures were fitted by several mathematical models. Specifically, a power-law, a diffusion-based and a combined diffusion-based model were evaluated to describe the accelerated thermal release of the active compounds. The mathematical models were developed based on the following assumptions:

- Samples are considered isotropic of similar shape and with equal initial active compound concentration.
- The effective diffusion coefficient through the samples is constant.
- There is no resistance to the mass transfer of the active compound from the external surfaces of the samples.
- The active compound is uniformly distributed in the samples.

The most generally accepted theory to model mass transport of a volatile active compound from a polymer matrix, is that based on Fick’s second law of diffusion, which in plane sheet geometry is described by Equation (4) [67]:

(4) $$\frac{\partial C}{\partial t}=D\left(\frac{\partial^{2}C}{\partial x^{2}}\right)$$

where C (mg kg^−1^) is the active compound concentration in the polymer, t (s) is time, D (cm^2^ s^−1^) is the diffusion coefficient, and x (cm) is the distance from the plane sheet. 

The temperature dependence of diffusion is governed by the activation energy required by the molecules to jump from one hole to another in the polymer matrix [67]. Therefore, the effect of temperature on diffusivity can be expressed by an Arrhenius function as in Equation (5):

(5) $$D=D_{0}e^{-\frac{E_{a}}{\mathrm{RT}}}$$

where D_0_ is a pre-exponential factor (cm^2^/s), E_a_ (kJ/mol) is the activation energy for diffusion (kJ/mol), R is the universal gas constant (kJ/mol × K) and T is the temperature in K.

Considering that the active compound (i.e., carvacrol or thymol) is evaporated at the surface of the polymer, the general solution for this diffusion problem (Equation (4)) in the form of the dimensionless ratio M_t_/M_∞_ and assuming surface evaporation from a plane sheet geometry, according to Crank is given by [67,68]:

(6) $$\frac{M_{t}}{M_{\infty }}=1-\overset{\infty }{\underset{n=1}{\sum }}\frac{2L^{2}\exp\left(-\beta_{n}^{2}\mathrm{Dt}/l_{p}^{2}\right)}{\beta_{n}^{2}\left\{\beta_{n}^{2}+L\left(L+1\right)\right\}}$$

where M_t_/$M_{\infty }$ (dimensionless) is the fractional mass of the active compound desorbed with time ($M_{\infty }$ is operationally defined as the total mass of the active compound in the polymer blend), l_p_ is the thickness of the polymer, L is the reciprocal of the mass transfer surface resistance ratio (given by L = (l_p_ k)/D with k being a proportionality constant in the equation relating the rate of material transport through the surface to the difference between the concentration on the polymer surface and the concentration in the gas phase [68]) and β_n_ are the positive roots of Equation (7) [67]:

(7) $$\beta_{n}{\tan\beta }_{n}=L$$

Values of several β_n_ (particularly the most important, i.e., from β_1_ to β_6_) for several values of L ranging from 0 to 100 are available in Tables in references [67] and [68]. 

Equation (6) can be used to simulate the experimental data from TGA measurements with the diffusion coefficient D as an adjustable parameter.

In the preceding analysis, D was assumed to depend only on temperature and not concentration. If the diffusion coefficient D varies with concentration, as is the case in many physical processes, then Equation (4) should be written in the following form [67]:

(8) $$\frac{\partial C}{\partial t}=\frac{\partial }{\partial x}\left(D\frac{\partial C}{\partial x}\right)$$

Equation (8) is much more difficult to solve and depends on the particular form providing the dependence of D on the concentration. Since this dependence is not known for a variety of systems, we preferred here to follow an approach also followed in a recent publication from our group, on the release of limonene from PMMA particles [60]. Accordingly, a two-component model was used to model the desorption of the active compound from the polymer sample. Thus, to take into account the dependence of D on concentration, two diffusion processes are considered to occur, a fast and a slow one characterized by a high and a low diffusion coefficient, respectively. Then, the total model equation comprising both the fast and the slow diffusivity of the desorbing active compound is given by [60].

(9) $${\frac{M_{t}}{M_{\infty }}|}_{\mathrm{total}}=\left(f\right){\frac{M_{t}}{M_{\infty }}|}_{\mathrm{fast}}+\left(1-f\right){\frac{M_{t}}{M_{\infty }}|}_{\mathrm{slow}}$$

where M_t_ (mg kg^−1^) is the total oil mass desorbed in time t (s) and *f* is the fraction of the fast desorption process. 

If Equation (6) for the two-compartment hypothesis is applied, then Equation (9) can be written as follows:

(10) $$\frac{M_{t}}{M_{\infty }}=f\left[1-\overset{\infty }{\underset{n=1}{\sum }}\frac{2L^{2}\exp\left(-\frac{\beta_{n}^{2}D_{f}t}{l_{p}^{2}}\right)}{\beta_{n}^{2}\left\{\beta_{n}^{2}+L\left(L+1\right)\right\}}\right]+\left(1-f\right)\left[1-\overset{\infty }{\underset{n=1}{\sum }}\frac{2L^{2}\exp\left(-\frac{\beta_{n}^{2}D_{s}t}{l_{p}^{2}}\right)}{\beta_{n}^{2}\left\{\beta_{n}^{2}+L\left(L+1\right)\right\}}\right]$$

According to Equation (10) a total of three parameters have to be estimated for each set of desorption data: the fast, D*_f_* and slow, D*_s_* diffusion coefficients and the fast desorption fraction (*f*). The fraction of the slow desorbing pool is calculated as (1 − *f*). 

Sometimes some of the basic assumptions made (e.g., isotropy, homogeneity or local equilibrium) break down in solid polymers. The result is that Fickian diffusion may not hold and non-Fickian expressions are often used. Generally, these types of expressions reduce to the following [69]:

(11) $$\frac{M_{t}}{M_{\infty }}={\mathrm{kt}}^{n}$$

where k is a constant depending on temperature and the exponent n characterizes the type of diffusion. Thus, a value of n equal to 0.5 denotes the classical Fickian diffusion, whereas values of n < ½ a pseudo-Fickian diffusion. If n exceeds ½ then the diffusion is characterized as anomalous and n = 1 denotes the so-called case II diffusion [69].

**Figure 10:**
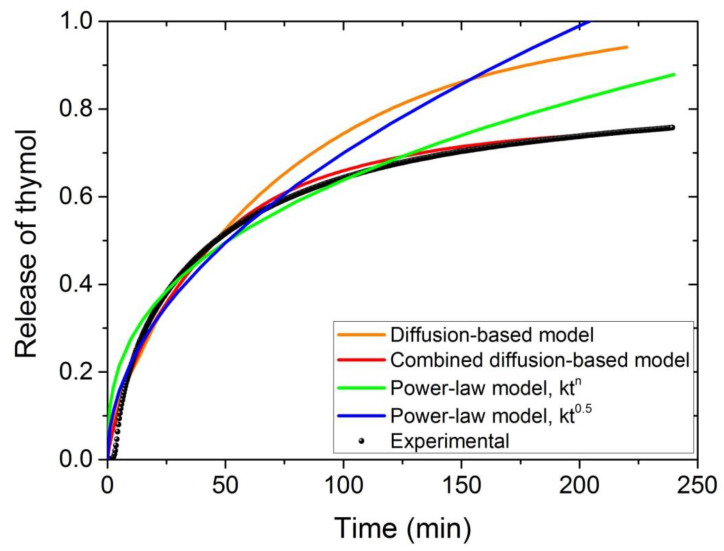
Comparison of different models in the simulation of the experimental data on the release profile of thymol at 142 °C. The diffusion model is based on Equation (6), the combined diffusion model on Equation (10) the power-law model on Equation (11), whereas in the last simulation (blue line) the same Equation (11) was used but setting the value of n fixed at 0.5.

In order to select the appropriate model to simulate the experimental data, the release of thymol was studied at 142 °C. Results from the diffusion-based model (Equation (6)) obtained after optimum fitting estimation of the diffusion coefficient seem to simulate the experimental data very well at the initial stage (until almost 0.5), whereas the values are overestimated at high mass release ratios (Figure 10). Similar behavior was found when the non-Fickian power law-model from Equation (11) was used and particularly very high values at large release times were observed when the exponent n was set fixed and equal to 0.5. It should be noticed that the best-fit value of the exponent n was found to be n = 0.365, meaning that the process is characterized as pseudo-Fickian diffusion. Finally, the simulation was excellent when the combined diffusion model was employed (red line in Figure 10. Of course, one could assume that this may be the result of using three adjustable parameters this time (i.e., *f*, D*_f_* and D*_s_*) instead of two in Equation (11) (i.e., k and n) and one when Equation (6) was used (i.e., D). However, this is partially true. The physical meaning is that, as the amount of the active compound released from the polymer matrix its concentration decreases significantly leading to different diffusivity compared to that estimated when high amounts of the active compounds are present. This explains the terms fast and slow diffusion associated with large and small amounts of the diffusant. 

**Figure 11:**
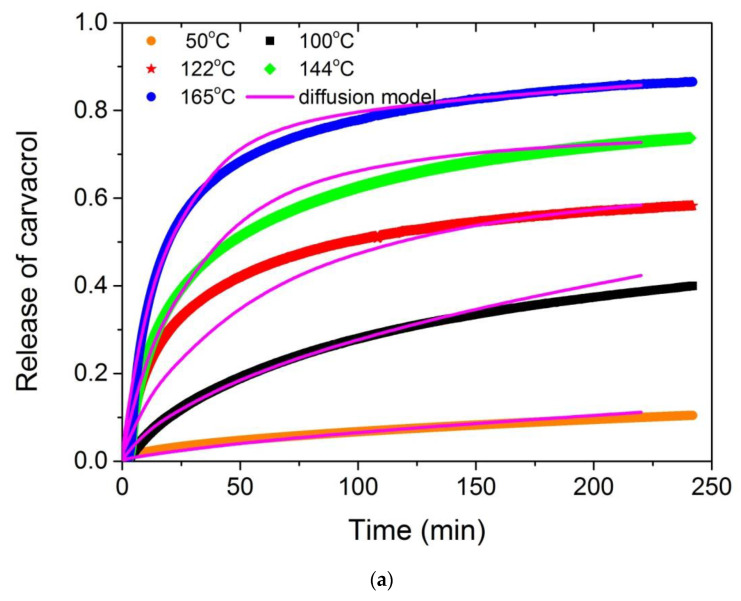

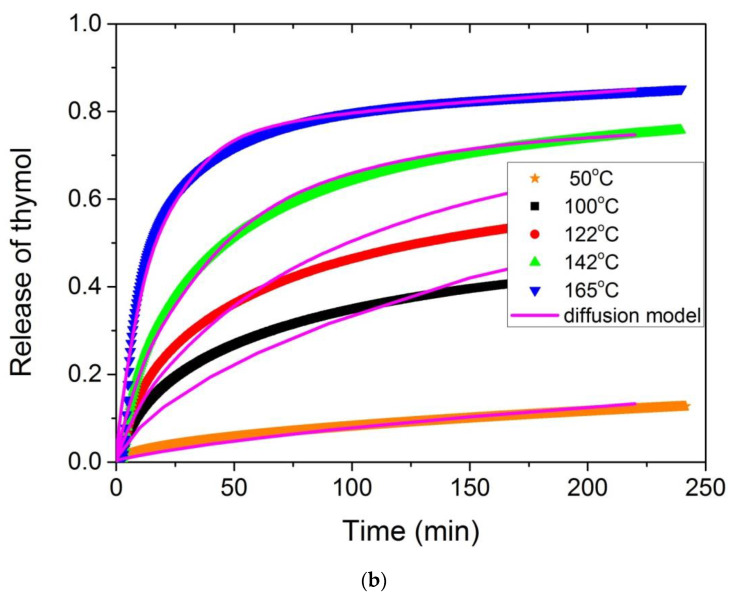
Comparison of the simulation results obtained from the combined diffusion model (Equation (10)) and the best-fit parameters shown in Table 4, to the normalized isothermal TGA experimental data plotted in the form of M_t_/M_∞_ and obtained at different temperatures, for carvacrol (**a**) and thymol (**b**).

Therefore, only the combined Equation (10) was used in all further experiments and the estimated optimum values of the adjustable parameters, *f*, D*_f_* and D*_s_* at different temperatures appear in Table 4. It can be seen that the values of the slow mode diffusion coefficient become very small at low temperatures, and it is for this reason that only their order of magnitude is included. As expected, an increase in temperature results in higher movement of the molecules and thus to higher diffusion coefficients. The values estimated for both thymol and carvacrol were found to be similar at a specified temperature, since the molecular structure of these compounds is also similar (Figure 1). Using these values of the parameters and the modelling Equation (10) the simulation curves are compared to the experimental data in the form of the dimensionless quantity M_t_/M_∞_ in Figure 11. As it is clear fitting is very good in most temperatures for both thymol and carvacrol release, whereas differences appear mainly at large release times. A similar good fit of experimental data using an equation similar to Equation (10) was also observed during the release of limonene [60].

**Figure 12:**
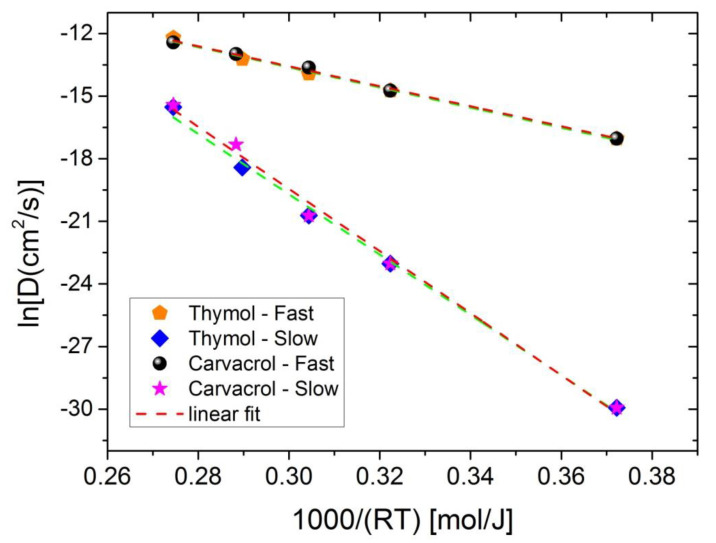
Arrhenius-type plots of the fast and slow diffusion coefficients for the release of thymol and carvacrol in order to estimate the activation energies of the processes.

Furthermore, the Arrhenius expression (Equation (5)) was used to calculate the activation energy of the diffusion process for both thymol and carvacrol. Typical Arrhenius-type plots appear in Figure 12 for both thymol and carvacrol release. Very good straight lines were obtained in all cases for both fast and slow diffusion. From the slope of the straight lines, the activation energy can be calculated, and the values are presented in Table 5. The activation energy of the fast diffusion process is near 48 kJ/mol for both thymol and carvacrol release, whereas a much larger value was calculated for the slow diffusion process, 145–150 kJ/mol.

### 3.4. Antioxidant Properties

**Figure 13:**
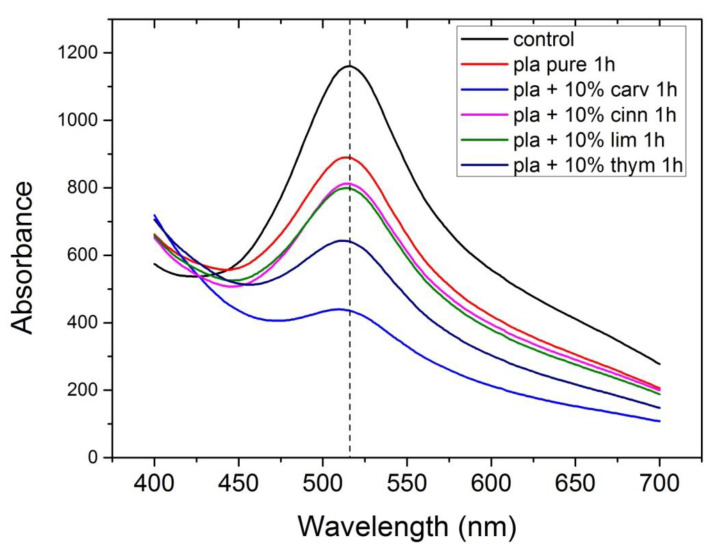
UV spectra of neat PLA and PLA composites with 10% of thymol, carvacrol, limonene and cinnamaldehyde after 1 h in DPPH solution.

**Figure 14:**
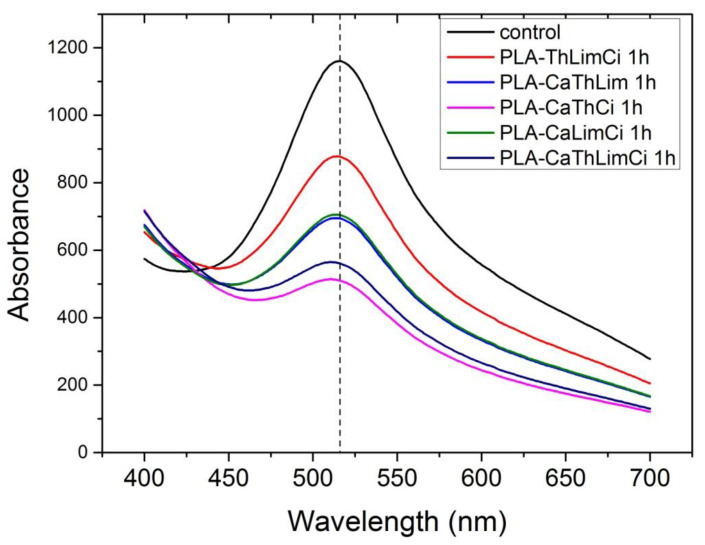
Comparative UV spectra of all ternary composites of PLA and the quaternary after 1 h in DPPH solution.

**Figure 15:**
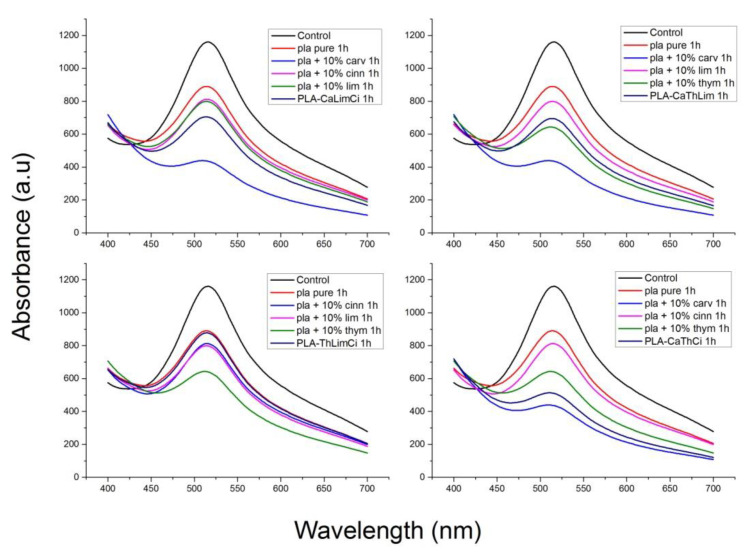
Comparative UV spectra of neat PLA, each ternary composite and the components of this composite after 1 h in DPPH solution.

**Figure 16:**
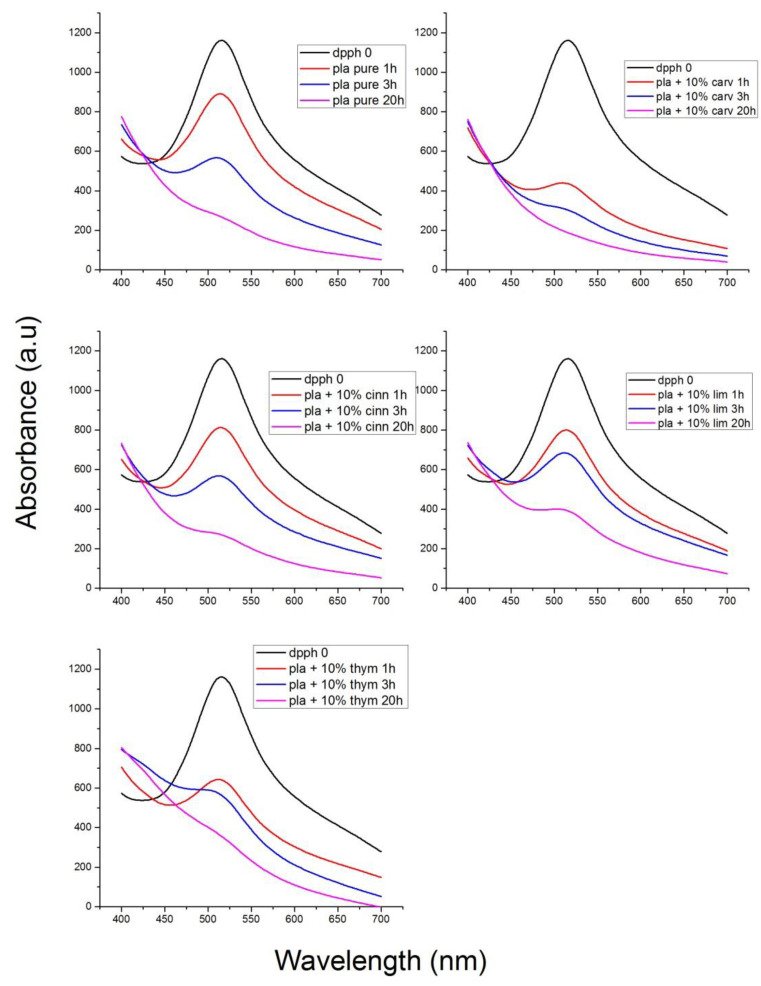
Effect of time stored in the DPPH solution of neat PLA and its composites with 10% of thymol carvacrol, limonene or cinnamaldehyde.

The antioxidant stability of the materials prepared was studied with the DPPH method. DPPH radical scavenging activity is one of the most frequently used methods to predict the antioxidant activity of packaging films [70]. The samples initially present a purple color. The change in color to yellowish is an indication of antioxidant activity of the additive. In order to quantify the change of the color, the UV absorbance of the samples was measured, and UV spectra of the materials studied appear in Figure 13, Figure 14, Figure 15 and Figure 16. 

**Figure 17:**
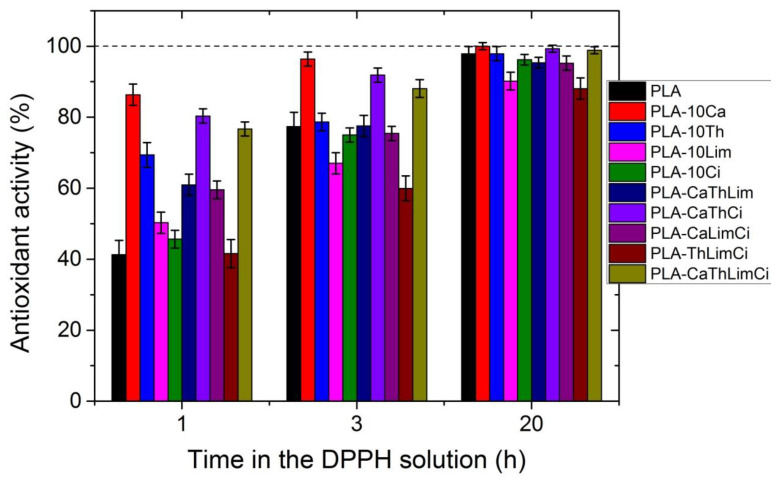
Antioxidant activity of all composites at several timepoints incubated in the DPPH solution.

In Figure 13, the UV spectra of neat PLA and PLA composites with 10% of thymol, carvacrol, limonene and cinnamaldehyde after 1 h in DPPH solution is illustrated. All materials exhibit a peak at 516 nm. The higher antioxidant activity was noticed when carvacrol was added, followed by thymol, whereas limonene and cinnamaldehyde presented a similar antioxidant activity. Interesting to point that neat PLA presented also some antioxidant activity. Furthermore, comparative UV spectra of the PLA composites with the triple and the quadruple mixtures, after 1 h in DPPH solution appear in Figure 14. Higher antioxidant ability was observed in the composite including both thymol and carvacrol, whereas the quaternary composite presented slightly lower antioxidant properties since the total amount of the more active compounds, i.e., thymol and carvacrol was lower than in the triple composite. As the amount of the additive was increased, the antioxidant ability was much increased. The effect of the antioxidant ability of each component in the ternary composites is illustrated in Figure 15. Finally, the UV spectra indicating the antioxidant activity as a function of the time remained in the DPPH solution was measured for all materials and result appear in Figure 16. It is seen that after 20 h the composites with thymol and carvacrol exhibit an almost 100% antioxidant ability. These results are quantified when estimating the antioxidant activity via Equation (2). Results for all the composites appear in Figure 17. The increase with time of the antioxidant activity is clear for all samples. Moreover, large differences are observed depending on the compound used. Thus, it is verified that carvacrol exhibits the higher antioxidant capacity followed closely by the composites with the triple mixture of carvacrol–thymol–cinnamaldehyde and the quadruple mixture.

## 4. Conclusions

In this research several bio-composites of poly(lactic acid), PLA, with ingredients found in thyme, oregano, citrus fruits and cinnamon, namely thymol, carvacrol, limonene and cinnamaldehyde were prepared and studied. Triple and quadruple blends of the active compounds in PLA were prepared using the solvent-casting technique in order to examine possible synergistic effects. The successful incorporation of the active ingredients into the polymer matrix was verified by FTIR measurement. XRD and DSC data revealed that the crystallinity of PLA was not significantly affected. However, the Tg of the polymer as measured by DSC was lowered, verifying the plasticization effect of all additives on the polymer matrix. Combination of additives resulted in much lower Tg values and therefore more intense plasticization. From thermal degradation measurements, it was found that cinnamaldehyde plays a catalytic role in the thermal degradation of PLA shifting the curves to slightly lower temperatures. Release of thymol or carvacrol from the composites is affected by temperature and time; at low temperatures, there is a constant but small amount of the active compound released. Treatment of these samples at high temperatures for prolonged time is not recommended since most of the active compound will be released in less than 1 day. A diffusion-based Fickian model, together with a combined diffusion and a power-law non-Fickian model were employed to study the release kinetics of thymol and carvacrol from the PLA films. It was found that a combined Fickian model including a fast and a slow process could simulate the experimental data very well on a wide range of temperatures. The corresponding fast and slow diffusivities were estimated, and the activation energies of the fast and slow diffusion process were found to be near 48 and 150 kJ/mol, respectively, for both thymol and carvacrol release. The higher antioxidant activity was noticed when carvacrol was added, followed by thymol, whereas limonene and cinnamaldehyde presented a similar lower antioxidant activity. It is interesting to point that neat PLA presented also some antioxidant activity. From the composites including the triple mixtures, the higher antioxidant activity was measured in that including thymol–carvacrol and cinnamaldehyde. The main conclusion of this study is that, comparatively to others, carvacrol-based composites present the higher antioxidant activity and better thermal properties. From the composites with the triple mixtures, best results as far as the properties of PLA measured here are obtained when a combination of thymol–carvacrol–cinnamaldehyde is used. It is not recommended to use more than three compounds simultaneously.

## Figures and Tables

**Table 1:** Experimental design. Amount of additive(s) in the PLA matrix and their codename.

Experiment #	Code	Thymol (wt%)	Carvacrol (wt%)	Limonene (wt%)	Cinnamaldehyde (wt%)
1	PLA	0	0	0	0
2	PLA-5Th	5	0	0	0
3	PLA-10Th	10	0	0	0
4	PLA-5Ca	0	5	0	0
5	PLA-10Ca	0	10	0	0
6	PLA-5Lim	0	0	5	0
7	PLA-10Lim	0	0	10	0
8	PLA-5Ci	0	0	0	5
9	PLA-10Ci	0	0	0	10
10	PLA-ThCaLim	3.33	3.33	3.33	0
11	PLA-ThCaCi	3.33	3.33	0	3.33
12	PLA-ThLimCi	3.33	0	3.33	3.33
13	PLA-CaLimCi	0	3.33	3.33	3.33
14	PLA-ThCaLimCi	2.5	2.5	2.5	2.5

**Table 2:** Quantification of the active ingredients in the PLA matrix.

Code	Preparation Method	Initial Loading of the Active Ingredient (wt%)	Loading Capacity (wt%)	Encapsulation Efficiency (%)
PLA		0	n.d.	0
PLA-5Th	Melt mixing	5	3.6 ± 0.1	72 ± 3
PLA-10Th	Melt mixing	10	6.8 ± 0.2	68 ± 2
PLA-5Th	Solution casting	5	4.9 ± 0.1	98 ± 2
PLA-10Th	Solution casting	10	9.5 ± 0.2	95 ± 2
PLA-5Ca	Solution casting	5	4.8 ± 0.1	97 ± 3
PLA-10Ca	Solution casting	10	9.6 ± 0.2	96 ± 2
PLA-5Lim	Solution casting	5	4.6 ± 0.1	92 ± 2
PLA-10Lim	Solution casting	10	9.0 ± 0.3	90 ± 3
PLA-5Ci	Solution casting	5	4.7 ± 0.2	94 ± 4
PLA-10Ci	Solution casting	10	9.7 ± 0.1	97 ± 1

**Table 3:** Thermal transitions, melting enthalpy and degree of crystallinity of PLA, and all composite films obtained by solution casting.

Material	T_g_ (°C)	ΔC_p_ (J/g·°C)	T_m1_ (°C)	T_m2_ (°C)	T_cc_ (°C)	ΔH_cc_ (J/g)	ΔH_f_ (J/g)	X_c_ (%)
PLA	58.4	0.43	147.4	155.1	107.7	26.34	27.53	29.6
PLA-10 Th	56.6	0.30	146.8	154.4	109.1	23.93	25.16	29.7
PLA-10 Ca	55.1	0.43	148.1	154.5	111.4	24.34	25.28	30.0
PLA-10 Lim	54.8	0.33	147.5	153.7	111.3	23.74	24.94	29.5
PLA-10 Ci	50.0		140.4	150.1	103.0	23.72	24.68	29.2
PLA-ThCaLim	56.2	0.24	146.5	154.2	107.5			
PLA-ThCaCi	52.3	0.34	146.7	152.0	104.9			
PLA-ThLimCi	49.5	0.58	147.5	--	111.3			
PLA-CaLimCi	54.2	0.54	147.0	152.8	109.0			
PLA-ThCaLimCi	49.4	0.34	145.6	--	107.4			

**Table 4:** Parameters estimated (i.e., fast, D*_f_* and slow, D*_s_* diffusion coefficients and fraction *f*) from fitting of the combined diffusion-based model (Equation (10)) to the experimental release data of thymol and carvacrol from the PLA films.

	Thymol				Carvacrol			
Temperature (°C)	D*_f_* (10^−6^ cm^2^/s)	D*_s_* (cm^2^/s)	*f*	R^2^	D*_f_* (10^−6^ cm^2^/s)	D*_s_* (cm^2^/s)	*f*	R^2^
50	0.04	10^−13^	0.72	0.997	0.04	10^−13^	0.60	0.988
100	0.4	10^−10^	0.72	0.948	0.4	10^−10^	0.60	0.968
122	0.9	10^−9^	0.72	0.939	1.2	10^−9^	0.60	0.962
142 *	1.8	10^−8^	0.73	0.991	2.3	3 × 10^−8^	0.68	0.986
165	5.0	1.8 × 10^−7^	0.72	0.992	4.0	2 × 10^−7^	0.72	0.989

* In the case of carvacrol, this temperature is 144°C.

**Table 5:** Estimated activation energies and pre-exponential factors for the fast and slow diffusion process during release of thymol or carvacrol.

	Ln (D_0_(cm^2^/s))	E_a_ (kJ/mol)	R^2^
Carvacrol			
Fast	0.805 ± 0.162	47.9 ± 1.5	0.9962
Slow	25.20 ± 2.03	148.8 ± 6.5	0.9925
Thymol			
Fast	0.866 ± 0.582	48.2 ± 1.8	0.9941
Slow	23.63 ± 1.67	144.4 ± 5.3	0.9946
